# BNP Signaling Is Crucial For Embryonic Stem Cell Proliferation

**DOI:** 10.1371/journal.pone.0005341

**Published:** 2009-04-28

**Authors:** Essam Mohamed Abdelalim, Ikuo Tooyama

**Affiliations:** 1 Molecular Neuroscience Research Center, Shiga University of Medical Science, Setatsukinowa-cho, Otsu, Japan; 2 Department of Cytology and Histology, Faculty of Veterinary Medicine, Suez Canal University, Ismailia, Egypt; University of Calgary, Canada

## Abstract

**Background:**

Embryonic stem (ES) cells have unlimited proliferation potential, and can differentiate into several cell types, which represent ideal sources for cell-based therapy. This high-level proliferative ability is attributed to an unusual type of cell cycle. The Signaling pathways that regulate the proliferation of ES cells are of great interest.

**Methodology/Principal Findings:**

In this study, we show that murine ES cells specifically express brain natriuretic peptide (BNP), and its signaling is essential for ES cell proliferation. We found that BNP and its receptor (NPR-A, natriuretic peptide receptor-A) were highly expressed in self-renewing murine ES cells, whereas the levels were markedly reduced after ES cell differentiation by the withdrawal of LIF. Targeting of BNP with short interfering RNA (siRNA) resulted in the inhibition of ES cell proliferation, as indicated by a marked reduction in the cell number and colony size, a significant reduction in DNA synthesis, and decreased numbers of cells in S phase. BNP knockdown in ES cells led to the up-regulation of gamma-aminobutyric acid receptor A (GABA_A_R) genes, and activation of phosphorylated histone (γ-H2AX), which negatively affects ES cell proliferation. In addition, knockdown of BNP increased the rate of apoptosis and reduced the expression of the transcription factor Ets-1.

**Conclusions/Significance:**

Appropriate BNP expression is essential for the maintenance of ES cell propagation. These findings establish BNP as a novel endogenous regulator of ES cell proliferation.

## Introduction

Embryonic stem (ES) cells have the remarkable capacity to divide indefinitely while retaining their wide range differentiation potential, and they represent a promising source for cell transplantation therapies [1]. They exhibit a very unusual cell cycle structure, characterized by a short G1 phase and a high proportion of cells in the S phase [2], [3], which is associated with a unique mechanism of cell cycle regulation.

Brain natriuretic peptide (BNP), a member of natriuretic peptide family, is produced predominately in the heart [4], [5], and recently, we have shown that BNP is expressed in ES cell-derived cardiomyocytes [6].The physiological effects of natriuretic peptides are initiated by binding to two particulate guanylate cyclase receptors; natriuretic peptide receptor type A (NPR-A), which is sensitive to ANP (atrial natriuretic peptide) and BNP [7], natriuretic peptide receptor type B (NPR-B), which is specific for CNP (c-type natriuretic peptide) [8] to produce intracellular cyclic guanosine monophosphate (cGMP) in response to hormone binding [7]. Natriuretic peptides regulate blood pressure and fluid homeostasis [9]. In addition, the abilities of natriuretic peptides to modulate cell growth and cell proliferation have received attention [10]. Cell-based studies have shown that ANP and BNP exhibit important autocrine and paracrine functions such as modulating myocyte growth, apoptosis and proliferation in smooth muscle cells [11]. BNP-transgenic mice exhibit overgrowth of the growth plate cartilage through a cGMP-dependent mechanism [12]. Furthermore, signaling through NPR-A has been found to play a pivotal role in tumor growth [13]. Although, little is known about the role of natriuretic peptides in pre-implantation embryo development, it has been reported that NPR-B-deficient mice were sterile due to lack of development of the reproductive system, and the majority (75%) of the NPR-B-deficient mice died before 100 days of age [14]. In addition, it has been found that exogenous BNP can enhance clonal propagation in murine ES cells [15], suggesting the presence of functional natriuretic peptide receptors in ES cells.

To date, there is no data available concerning the expression of BNP in ES cells. Therefore, in the present study we have characterized the expression of BNP in undifferentiated ES cells, and examined its role in regulating ES cell proliferation. We found that BNP and its receptor NPR-A are specifically expressed in self-renewing ES cells, and the BNP signaling plays an important role in maintaining the proliferation of ES cells by inhibiting GABA_A_R and Ets-1 genes.

## Results

### Expression of BNP and its receptors in pluripotent ES cells and pre-implantation embryos

**Figure 1:**
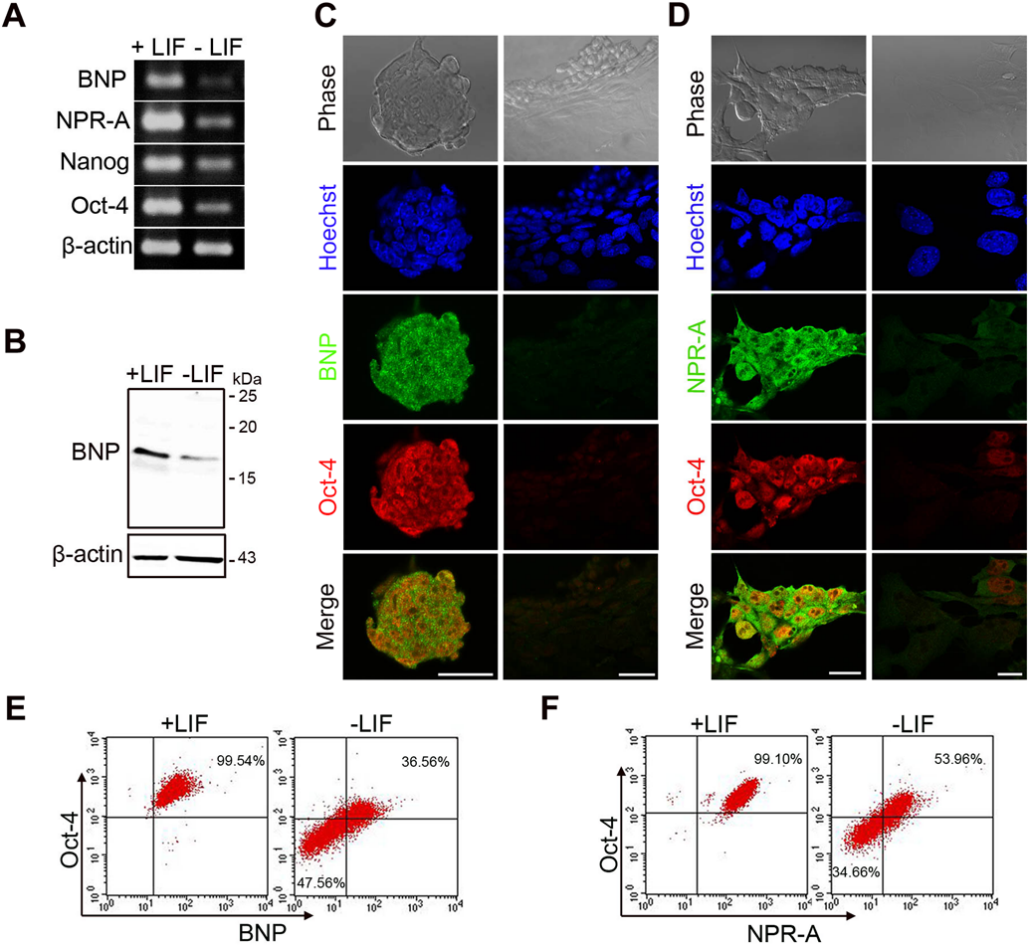
BNP and NPR-A are expressed specifically in self-renewing ES cells. [A] RT-PCR analysis showing reductions in the levels of BNP and NPR-A mRNA after culture with (+) LIF in the ES cell medium or without (−) LIF for 5 days in the differentiation medium. Oct-4 and nanog were used as self-renewal markers, and β-actin was used as a loading control. [B] Western blot showing down-regulation of the BNP protein after LIF removal in ES cells treated as in A. β-actin was used as a loading control. [C] Double-immunofluorescence images of ES cells treated as in A, stained with antibodies against the ES cell marker Oct-4 and BNP, and counterstained with Hoechst reagent. [D] Immunofluorescence images of ES cells treated as in A, stained with antibodies against Oct-4 and NPR-A, and counterstained with Hoechst reagent. Note that the BNP and NPRA signals are down-regulated upon differentiation. [E] Flow cytometric analysis of cells treated as in A, showing the expression of BNP and Oct-4 in pluripotent ES cells and differentiated ES cells. [F] Flow cytometric analysis of cells treated as in A, showing the expression of NPR-A and Oct-4 in pluripotent ES cells and differentiated ES cells. Scale bars in C, D = 20 µm.

Initially, we examined the expression of BNP and its receptor, NPR-A, in murine ES cells grown under self-renewal and differentiation conditions (Fig. 1). Polymerase chain reaction with reverse transcription (RT-PCR) (Fig. 1A), Western blotting (Fig. 1B), double-immunofluorescence (Fig. 1C, D), and flow cytometry (Fig. 1E, F) analyses showed that BNP and NPR-A were highly expressed in pluripotent ES (Oct-4-positive) cells that were cultured in the presence of LIF, and that expression was down-regulated upon differentiation induced by culturing ES cells without LIF for 5 days (Fig. 1). The differentiated cells, which were negative for oct-4 expression, were also negative for BNP and NPR-A expressions (Fig. 1C, D). These results indicate that BNP and NPR-A are specifically expressed in self-renewing ES cells.

**Figure 2:**
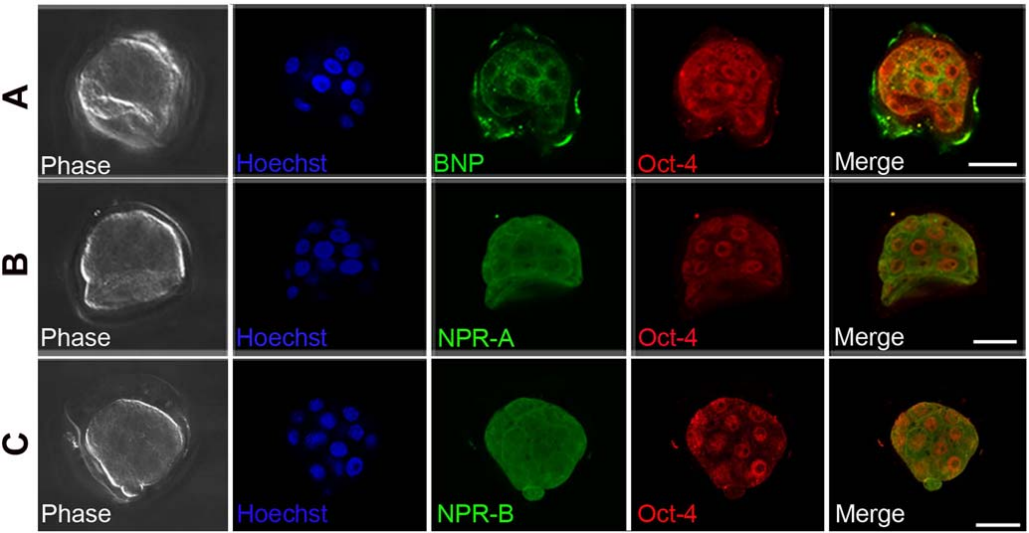
BNP and its receptors are expressed in pre-implantation embryos. Double-immunofluorescence images of 3.5-day-old blastocysts stained with antibodies against BNP and Oct-4 [A], NPR-A and Oct-4 [B], or NPR-B and Oct-4 [C], and counterstained with Hoechst reagent. Scale bars = 20 µm.

To determine whether BNP, NPR-A and NPR-B are expressed in pre-implantation embryos, 3.5-day-old murine blastocysts were subjected to double-immunofluorescence analysis using anti-BNP, anti-NPR-A, or anti-NPR-B antibodies, as well as an antibody against Oct-4 (self-renewal marker). Consistent with their presence in the undifferentiated ES cells, BNP, NPR-A and NPR-B were co-expressed with the self-renewal regulator Oct-4 in the blastocyst (Fig. 2A–C). These findings suggest that BNP exerts regulatory functions prior to embryo implantation and in undifferentiated ES cells.

### BNP signaling regulates ES cell proliferation

**Figure 3:**
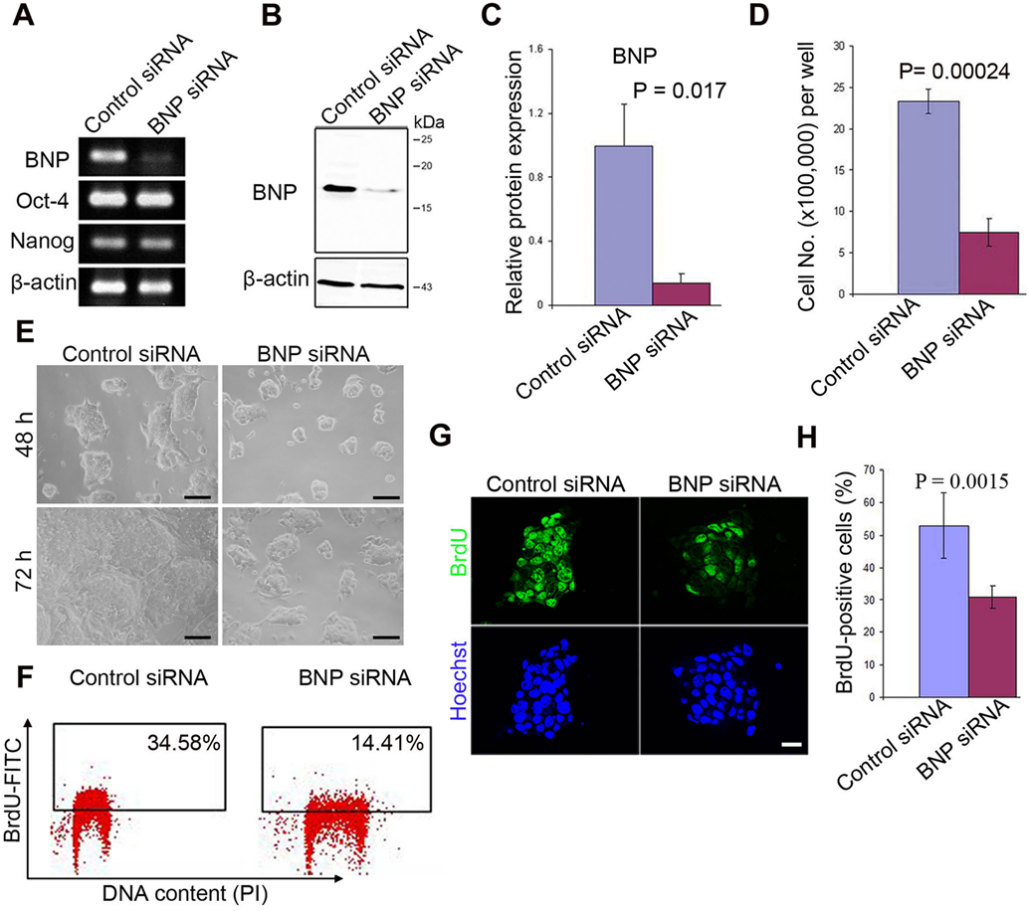
Knockdown of BNP suppresses ES cell proliferation. [A] RT-PCR analysis of ES cells transfected with the control siRNA or BNP siRNA, showing knockdown of the BNP gene 48 h after siRNA transfection. siRNA-mediated knockdown of BNP has no effect on the self-renewal marker genes (Oct-4 and nanog). β-actin was used as the internal control. [B] Western blot analysis of ES cells treated as in A, showing a reduced level of BNP protein 48 h after siRNA transfection. β-actin was used as a loading control. [C] Quantification of B (n = 3). [D] Quantification of ES cells 72 h after siRNA transfection (n = 3). [E] Morphologies of murine ES cells 48 h and 72 h after transfection with the control siRNA or BNP siRNA. [F] Flow cytometric analysis of BrdU incorporation in ES cells treated as in A. X-axis, DNA content, as shown by propidium iodide (PI) binding; y-axis, BrdU uptake after 45 min of exposure. [G] Immunofluorescence of BrdU incorporation (45 min) 48 h after transfection with the control siRNA or BNP siRNA. [H] Percentage of BrdU-positive nuclei in A (*n* = 30 fields). Data represent mean±s.d (p-value from two-tailed Student's *t*-test). Scale bars, 100 µm (E), 20 µm (G).

BNP expression in undifferentiated ES cells and repression upon differentiation suggested that it may play a role in maintaining pluripotency. We investigated this possibility by examining the effect of BNP knockdown in ES cells. A small interfering RNA (siRNA)-based technique was employed to specifically knockdown the BNP gene in undifferentiated ES cells that were maintained in a feeder-free culture. Before siRNA transfection, the undifferentiated status of the murine ES cells used for transfection was confirmed by flow cytometry and immunofluorescence (for Oct-4-positive cells). RT-PCR revealed a marked reduction in the level of BNP mRNA at 48 h post-transfection in the ES cells that were transfected with BNP-targeting siRNA (BNP siRNA), as compared with the ES cells that were transfected with a non-targeting siRNA (control siRNA) (Fig. 3A). This finding was confirmed by the reductions in protein levels observed in Western blots (Fig. 3B, C). Knockdown of BNP significantly reduced the number and colony size of the ES cells, as compared with the ES cells that were treated with the control siRNA (Fig. 3D, E).

To validate further the observed decrease in ES cell proliferation after BNP knockdown, siRNA-transfected cells were exposed to bromo-deoxyuridine (BrdU), and its incorporation into ES cells was quantified by immunofluorescence and flow cytometry. ES cells were examined 48 h post-transfection and following pulsed incorporation of BrdU (45 min). Flow cytometric analysis of BrdU, which was performed concurrent with the analysis of the cellular DNA content, showed a marked reduction in BrdU incorporation in ES cells treated with BNP siRNA (Fig. 3F). Likewise, we observed a significant reduction in the number of BrdU-positive cells in the BNP siRNA-treated cells, as compared to the control siRNA-treated cells, as shown by immunofluorescence (Fig. 3G, H). These results suggest that knockdown of BNP signaling has a significant effect in decreasing the rate of DNA synthesis.

**Figure 4:**
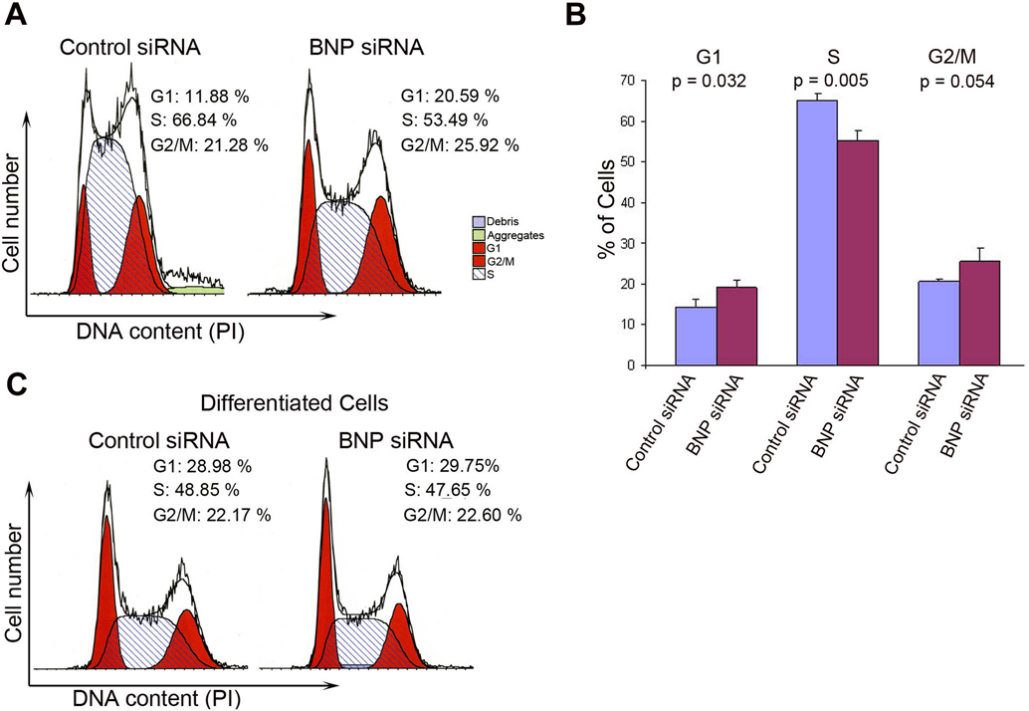
Effect of BNP Knockdown on ES cell cycle in murine ES cells. [A] Cell cycle distribution of ES cells 48 h after transfection with control siRNA or BNP siRNA. [B] Percentages of cells in G1, S, and G2/M phases of cell cycle (n = 3). [C] Cell cycle analysis of differentiated ES cells (6 days without LIF), 48 h after transfection with the control siRNA or BNP siRNA show no significant difference after BNP knockdown..

To assess directly the effect of BNP knockdown on the cell cycle profile, we analyzed the phases of the cell cycle 48 h after siRNA transfection. There was a significant reduction in the proportion of cells in S phase, and an increase in the proportion of cells in G1 and G2/M phases in ES cells treated with BNP siRNA (Fig. 4A, B). In contrast, there were no significant changes in the cell cycle profile of differentiated ES cells after BNP knockdown (Fig. 4C). These results suggest that inhibition of endogenous BNP suppresses DNA synthesis as a result of the reduction of the cells in the S phase in undifferentiated ES cells.

Furthermore, we examined the effect of exogenous BNP on ES cell proliferation. The presence of NPR-A in ES cells (Fig. 1A, D), suggests that BNP signaling can be transduced in ES cells *via* NPR-A. BNP addition to ES cells in low-density ES cell cultures, as previously reported [15], resulted in increased ES cell proliferation, as evidenced by the ES cell morphology (Supplemental Fig. 1A). However, the proliferative potency of exogenous BNP appeared to be lower than that of endogenously produced BNP, suggesting that the BNP/NPR-A signaling produced by ES cells masks the effect of exogenous BNP on ES cell proliferation.

### BNP knockdown and exogenous BNP have no effect on the ES cell pluripotent markers

**Figure 5:**
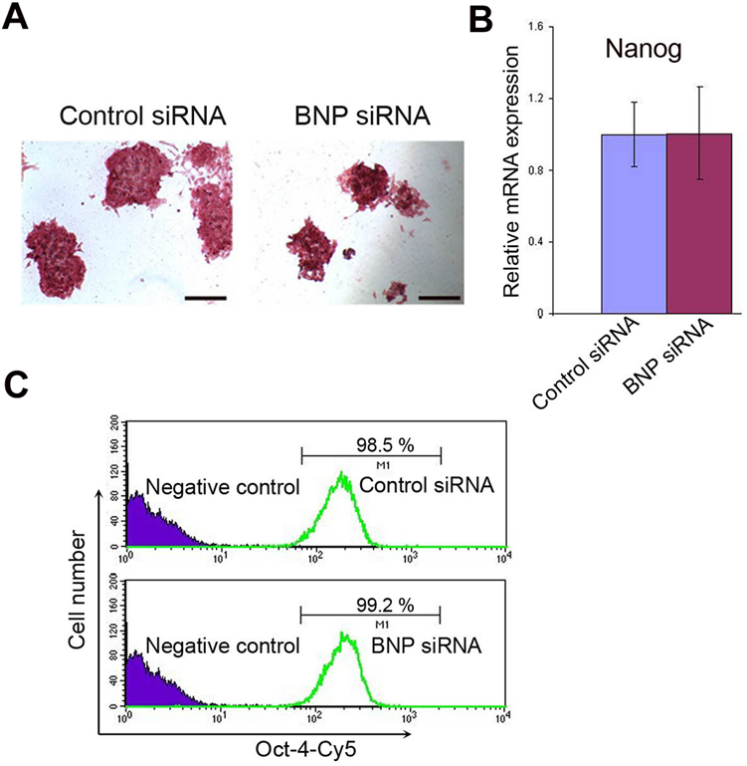
BNP knockdown does not affect on the ES cell pluripotency. [A] Alkaline phosphatase staining of ES cells 4 days after transfection with control siRNA or BNP siRNA. [B] Real-time PCR analysis shows equal level of nanog mRNA (pluripotent marker) in ES cells 48 h after siRNA transfection with control siRNA or BNP siRNA (n = 3). [C] Flow cytometric analysis of Oct-4 expression (a marker of pluripotency) in ES cells treated as in A, shows no significant difference in Oct-4 levels after BNP knockdown. Data represent mean±s.d (p-value from two-tailed Student's *t*-test). Scale bars, 10 µm (F).

The reduction in ES cell proliferation caused by abrogation of BNP signaling had no effect on the undifferentiated status of the ES cells, as determined by morphologic examination (Fig. 3E) and confirmed by measurements of alkaline phosphatase activity, which revealed that the levels of self-renewal in the control siRNA- and BNP siRNA–treated cells were identical (Fig. 5A). In addition, examination of the pluripotent ES cell markers Oct-4 and nanog 48 h post-transfection confirmed these results (Fig. 3A, 5B, C). Thus, ES cells treated with BNP siRNA expressed nanog mRNA (Fig. 3A, 5B) and Oct-4 mRNA and protein (Fig. 3A, 5C) at levels equivalent to those in ES cells treated with the control siRNA. Also, addition of BNP to ES cell cultures promotes ES cell propagation (Fig. S1A) without loss of pluripotent markers, as assessed by alkaline phosphatase activity 4 days after BNP addition (Fig. S1B). These findings indicate that reducing BNP does not affect ES cell pluripotent markers, and underline the role of BNP in undifferentiated murine ES cells rather than in spontaneously differentiated progeny.

### BNP knockdown suppresses cGMP production and NPR-B mRNA

**Figure 6:**
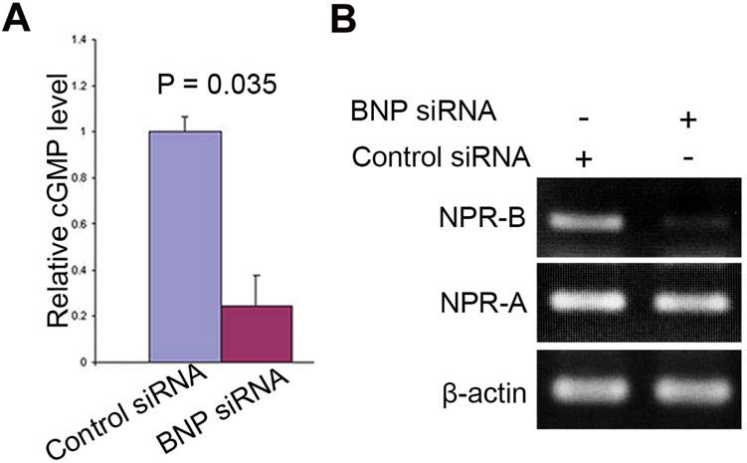
siRNA-mediated knockdown of BNP reduces cGMP production and NPR-B mRNA. [A] cGMP assay of ES cells 48 h after ES cell transfection with the control siRNA or BNP siRNA. [B] PCR analysis of NPR-A and NPR-B 48 h after ES cell transfection with the control siRNA or BNP siRNA . Data represent mean±s.d (n = 3; p-value from two-tailed Student's *t*-test).

In addition, we measured the intracellular levels of cGMP, to determine whether the cGMP pathway is involved in the effects of BNP knockdown on ES cells. The levels of cGMP were reduced significantly in ES cells treated with BNP siRNA, as compared to the levels in ES cells treated with the control siRNA (Fig. 6A). It is known that the natriuretic peptides -induced cGMP production is mainly due to the activation of NPR-A and/or NPR-B. Examination of NPR-A and NPR-B genes showed that NPR-B mRNA was markedly reduced in ES cells treated with BNP siRNA, as compared to the ES cells treated with the control siRNA, and no change was observed in the levels of NPR-A mRNA (Fig. 6B). These results suggest that the cGMP reduction was mainly due to the suppression of NPR-B gene.

### Knockdown of BNP signaling up-regulates GABA_A_R genes

**Figure 7:**
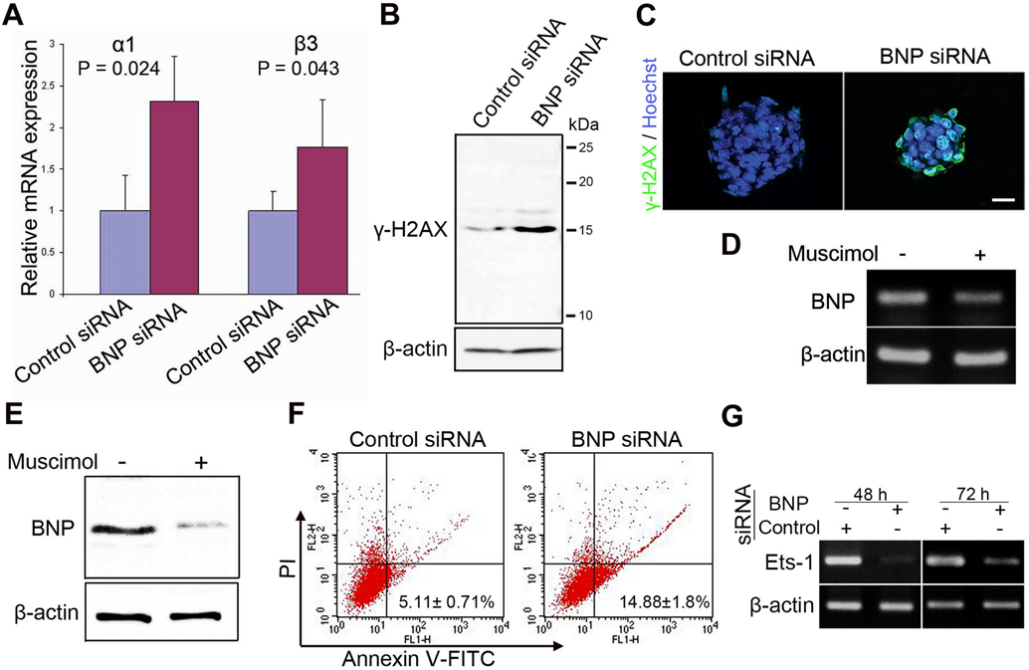
BNP knockdown upregulates GABA_A_R genes, induces apoptosis and downregulates Ets-1 in murine ES cells. [A] Real-time PCR analysis of the mRNA levels for the GABA_A_R α1 and GABA_A_R β3 subunits 48 h after ES cell transfection with the control siRNA or BNP siRNA. [B] ES cells treated as in A were analyzed by Western blotting using the anti-γ-H2AX antibody. β-actin is shown as a control for loading. [C] Immunofluorescence analysis shows the distribution of γ-H2AX foci (green) in ES cells treated as in A and B. DNA was counterstained with Hoechst reagent (blue). [D] PCR analysis of ES cells for BNP expression after 24 h of exposure to muscimol. [E] Western blot analysis of BNP in ES cells treated as in D. [F] Apoptosis assay 48 h after transfection with control siRNA or BNP siRNA. Flow cytometry profile represents Annexin-V-FITC staining in *x* axis and PI in *y* axis. The number represents the percentage of early apoptotic cells in each condition (lower right quadrant) (n = 2; p-value = 0.016). [G] PCR analysis of Ets-1 mRNA 48h and 72 h after ES cell transfection with the control siRNA or BNP siRNA. Data represent mean±s.d (n = 3; p-value from two-tailed Student's *t*-test).

To elucidate the mechanisms underlying the effects of BNP signaling on ES cell proliferation, we used Real-Time PCR to analyze the expression level of GABA_A_R 48 h after siRNA transfection. We found that the expression levels of the GABA_A_R α1 and β3 subunits, which are the major units of GABA_A_R in ES cells, were significantly up-regulated (Fig. 7A) in response to the suppression of BNP in ES cells.

To determine whether the elevated levels of the GABA_A_R subunits in BNP-knockdown ES cells are associated with the activation of γ-H2AX, as previously described [16], we examined the level of γ-H2AX by Western blotting. As expected for the presence of GABA_A_R up-regulation, γ-H2AX was expressed more abundantly in ES cells treated with BNP siRNA than in those treated with control siRNA (Fig. 7B). Furthermore, immunofluorescence analysis revealed the accumulation of γ-H2AX nuclear foci in response to BNP knockdown (Fig. 7C).

Furthermore, activation of endogenous GABA_A_R with muscimol (a GABA_A_R agonist) in low-density cultures, significantly reduced the BNP mRNA (Fig. 7D) and protein (Fig. 7E) levels, which are associated with reductions in ES cell proliferation, suggesting a regulatory mechanism between endogenous BNP and GABA_A_R signaling for the control of ES cell proliferation.

### Knockdown of BNP signaling induces apoptosis and down-regulates Ets-1

The reduced cell numbers and colony size that we observed in ES cells after BNP knockdown could be caused by increased cell death and/or decreased cell proliferation. Therefore, the apoptosis assay was performed using Annexin V as a marker for apoptotic cells. Flow cytometric analysis showed that the percentage of cells undergoing apoptosis significantly increased in the BNP siRNA-treated cells, as compared to the control siRNA-treated cells (Fig. 7F). These data indicate that BNP plays an important role in maintaining ES cell survival. However, it is known that GABA_A_R activation does not cause apoptosis or DNA damage in ES cells. Therefore, we examined the phosphoinositide 3-kinase (PI3K) pathway, which is known to be involved in proliferation and growth of ES cells by stimulating the G1-S phase transition, and its inhibition induces apoptosis in ES cells [17] **.** Western blot (Fig. S2A–B) and flow cytometry (Fig. S2C) analyses revealed no change in the level of phospho-Akt (ser 473), a marker for PI3K, suggesting that BNP signaling does not interfere with PI3K pathway. Furthermore, we examined the mRNA levels of the transcription factor Ets-1, which is required for normal survival of cells. Using RT-PCR, we found that the expression levels of the Ets-1 significantly down-regulated (Fig. 7G) at 48 h and 72 h from transfection in ES cells treated with BNP siRNA, indicating that BNP is required for the activation of Ets-1 in ES cells.

## Discussion

In the present study we demonstrate for the first time that undifferentiated murine ES cells express BNP and its receptor. We also report that BNP signaling is essential for murine ES cell survival and their clonal growth, through the suppression of GABA_A_R genes and activation of the transcription factor Ets-1. Together, these findings establish BNP as a novel regulator for murine ES cell proliferation.

The results showed that the expression of BNP and NPR-A were observed only in Oct-4-positive cells in the undifferentiated ES cells and the pre-implantation embryos. These results establish definitively that BNP and NPR-A are specifically expressed in self-renewing ES cells. During development, Oct-4 expression is required to maintain the pluripotent cell population of the inner cell mass (ICM) and epiblast. In addition, Oct-4 is highly expressed in ES cells, and without this factor, these cells differentiate along the trophoblast lineage [18].These findings suggest that BNP may exert regulatory functions prior to embryo implantation and in undifferentiated ES cells. Therefore, we investigated this possibility by examining the effect of BNP knockdown in ES cells, which significantly suppressed ES cell proliferation by decreasing the percentage of cells in S phase (DNA synthesis phase) and accumulation of cells in G1 and G2/M phases. The mechanism underlying the accumulation of cells in G2/M cell cycle phases is unknown, which suggest that BNP may has a role in regulating G2/M cell cycle transition, as well as G1/S in ES cells. These findings suggest the important role of endogenous BNP in maintaining the ES cell proliferation.

BNP is known to play a role in cell growth [12], [15]. A transgenic mouse over-expressing BNP exhibited marked skeletal overgrowth, and studies using in vitro organ culture of mouse tibia demonstrated that BNP increases cGMP production and activates the proliferation of growth plate chondrocytes via GC-coupled natriuretic peptide receptors [12]. In contrast, no skeletal defects are reported in transgenic mice overexpressing ANP [19], although ANP and BNP have similar affinity to known GC-coupled natriuretic peptide receptors [8], [20]. It is interesting to note mice with targeted deletion of BNP [21] exhibit a different phenotype than ANP-deficient mice [22]. Mice without BNP do not have hypertension; instead they show focal ventricular fibrosis [21]. This had led to the speculation that there may be a separate unknown receptor for BNP in cardiac fibroblasts.

However, in cultures of embryonic mouse tibias, BNP and CNP increased bone growth and stimulated cGMP production by signaling through NPR-B [23]. Moreover, Mice lacking NPR-A exhibit cardiac hypertrophy, hypertension, fibrosis [24]. These findings agree with our results, which showed that BNP knockdown in ES cells led to a marked decrease in intracellular cGMP level and a significant reduction in mRNA level of NPR-B. It is possible that the reduced cGMP level in ES cells is a reflection of the reduced NPR-B mRNA level. This hypothesis represents the signaling pathway through NPR-B as a possible regulator for ES cell proliferation. However, further studies are needed to elucidate the role of NPR-B in ES cell proliferation. It has been reported that mice lacking NPR-B were sterile due to lack of development of the reproductive system [14]. Furthermore, in the current study NPR-B expression was detected in Oct-4-positive cells of the pre-implantation embryos, suggesting a role of NPR-B in early stages of development. Taken together, these findings suggest that BNP could be exerting its proliferative effects through either NPR-A, its preferred receptor or through NPR-B, for which it has reasonable affinity [25].

BNP knockdown led to up-regulation of GABA_A_R genes. Furthermore, the GABA_A_R activation led to suppression of BNP expression in ES cells. Recently, the α1 and β3 subunits have been found to be the major subunits of GABA_A_Rs in ES cells, and they negatively regulate ES cell proliferation through a phosphorylated histone (γ-H2AX)-dependent mechanism [17]. However, the correlation between BNP and GABA_A_R has been previously determined, whereby BNP suppresses GABA_A_R currents in retinal bipolar cells [26]. These findings suggest that endogenous BNP signaling is important for inhibiting the GABA_A_R levels in ES cells, which in turn promotes ES cell proliferation.

Also, we found that BNP knockdown led to accumulation of γ-H2AX in ES cells. H2AX is considered to be critical for the surveillance of genome integrity [27]. It was found that GABA_A_R activation led to phosphorylation of H2AX in cell cycle-dependent and DNA damage-independent manners [16], [28]. However, our results showed an increase in the rate of apoptosis in the BNP siRNA-treated cells. Taken together, these findings suggest that accumulation of γ-H2AX after BNP knockdown in ES cells may be as a result of the activation of GABA_A_Rs and/or apoptosis.

BNP knockdown in ES cells led to a marked reduction in the transcription factor Ets-1. Ets-1 was shown to be required for the normal survival of T cells, and Ets-1^−/−^ T cells displayed a severe proliferative defect and demonstrated increased rates of spontaneous apoptosis, indicating anti-apoptotic role [29], [30]. In addition, Ets-1 was reported to participate in the activation of BNP gene [31]. Furthermore, NPR-A gene transcription and guanylyl cyclase (GC) activity of the receptor are critically regulated by Ets-1 in target cells [32]. Although, there is no data available about the role of Ets-1 in ES cell proliferation, these results indicate that the Ets-1 may be involved in the anti-apoptotic and proliferative effects of endogenous BNP on ES cells.

**Figure 8:**
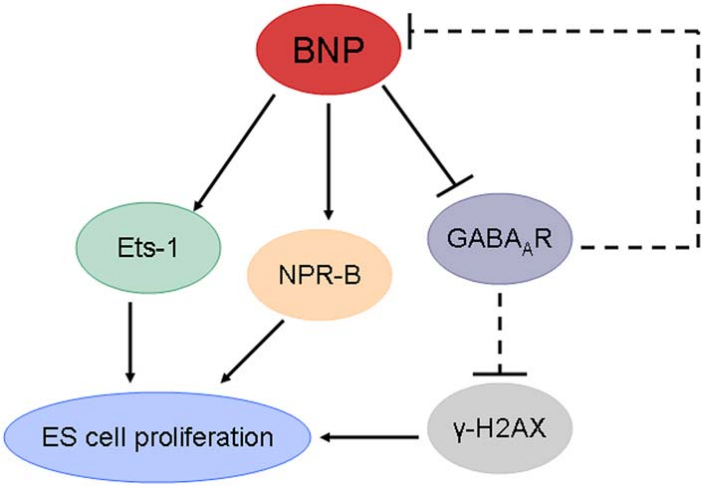
Proposed model of BNP-mediated maintenance of ES cell proliferation. BNP suppresses GABA_A_R, which in turn suppresses the phosphorylation of H2AX into γ-H2AX. As a result, activation of ES cell proliferation occurs. Also, the activation of GABA_A_R leads to suppression of BNP. In other pathways, BNP activates NPR-B and Ets-1, which may enhance the proliferation and survival of ES cells.

In summary, our data establish BNP as an essential regulator for the proliferation of murine ES cells. The undifferentiated ES cells express high levels of BNP and its specific receptor. Knockdown of BNP in ES cells led to a marked decrease in ES cell proliferation through a cGMP-dependent mechanism. The reduction of ES cell number observed in BNP siRNA-treated cells was primarily due to GABA_A_R activation, γ-H2AX accumulation, Ets-1 inhibition and apoptosis induction (Fig. 8). The present study defines a new pathway that is mediated by BNP-GABA_A_R for control ES cell propagation in vitro (Fig. 8). These findings will facilitate our understanding of the signaling pathways that maintain the unusual proliferative characteristics of ES cells.

## Materials and Methods

### ES Cell Culture

Murine ES cells (E14TG2a) (CRL-1821; American Type Culture Collection, Manassas, VA, USA) were maintained in DMEM/F-12 medium (Sigma) that was supplemented with 1000 U/ml LIF (Chemicon), 11% FBS, 2 mM glutamine (Nacalai Tesque, Japan), 1 mM sodium pyruvate (Sigma), 1% MEM nonessential amino acids (GIBCO), 0.1 mM 2-mercaptoethanol (Sigma) and 1% penicillin-streptomycin. For siRNA transfection, ES cells were cultured in the same medium, except that FBS was replaced with 15% Knockout Serum Replacement (KSR; GIBCO). Murine ES cells were cultured under feeder-free conditions in the presence of LIF. For differentiation, embryoid bodies were grown in bacteriologic dishes without LIF and β-mercaptoethanol for 5 days, as described previously [33]. Murine BNP (American Peptide) and muscimol (Tocris) [16] were added at 1 µM and 100 µM, respectively, to the cultured ES cells, while the control cells received vehicle alone.

### Immunofluorescence

ES cells grown on glass coverslips were rinsed briefly with PBS and fixed for 20 min in 4% paraformaldehyde in 0.1 M phosphate buffer (pH 7.4). Blastocysts were collected from ICR mice at 3.5 dpc and fixed in 4% paraformaldehyde in 0.1 M phosphate buffer (pH 7.4). All experimental procedures and protocols were reviewed and approved by the Institutional Animal Care and Use Committee of the Shiga University of Medical Science, Otsu, Japan and conformed to the NIH Guide for the Care and Use of Laboratory Animals.

The ES cells and blastocysts were permeabilized for 10 min with 0.1% Triton X-100 in PBS, and blocked for 40 min with 4% BSA in PBS at room temperature (RT). They were then incubated at 4°C overnight with the following antibodies; anti-BNP (1∶1000; Chemicon), anti-NPR-A (1∶300; Abcam), anti-NPR-B (1∶200, Abcam), anti-Oct-4 (1∶200, sc-5279; Santa Cruz Biotechnology), and anti-γ-H2AX IgG (1: 500; Upstate). This was followed by incubation with the following secondary antibodies; Chromeo 546-labelled anti-mouse IgG (1∶1000; Active Motif Chromeon GmbH, Tegernheim, Germany), Alexa Fluor 488-labeled anti-mouse IgG, and Alexa Fluor 488-labeled anti-rabbit IgG (1∶500; Molecular Probes). Nuclei were counterstained with Hoechst 33342 (1 µg/ml) (Invitrogen). The slides were examined by confocal laser microscopy (C1si; Nikon, Japan) and the images were processed using the Nikon EZ-C1 viewer software.

Immunofluorescence analysis of BrdU incorporation was performed as described previously [34]. BrdU (1∶100; Invitrogen) was added to the culture medium 48 h after transfection, and the incubation was continued for an additional 45 min. Cells were incubated with Alexa Fluor 488-conjugated mouse anti-BrdU antibody (1∶200; Molecular Probes) in 2% BSA-PBS overnight at 4°C. The percentage of BrdU-positive nuclei relative to the total number of nuclei per field of vision was determined. A minimum of five fields of vision per coverslip was counted.

### Reverse Transcription-Polymerase Chain Reaction (PCR) and Real-Time PCR

Total RNA was extracted using the RNeasy Mini kit (Qiagen) according to the manufacturer's instructions. cDNA synthesis was performed with 1 µg of total RNA using the Superscript III first-stand cDNA synthesis kit (Invitrogen). The cDNA (0.5 µg) was used as a template in a mixture that contained Ampli Taq Gold polymerase (Applied Biosystems). RT-PCR was performed with an annealing temperature of 58°C.

The cDNA samples were analyzed by Real-Time PCR in a LightCycler Real-Time PCR system (Roche Diagnostics) using the SYBR Premix Ex Taq II (Takara, Japan). Standard curves were generated for each Real-Time PCR run using serial 5-fold dilutions of samples that contained the gene of interest. Following PCR, the specificities and identities of the RT-PCR products were verified using melting curve analysis; this analysis distinguished specific PCR products from non-specific PCR products that arose from primer-dimer formation. The reactions were carried out in triplicates. The relative mRNA levels were determined from the appropriate standard curves and corrected for the β-actin mRNA levels.

The primer sequences (forward and reverse) were as follows: BNP, 5′-CTGAAGGTGCTGTCCCAGATG-3′ and 5′- GACGGATCCGATCCGGTC-3′; NPR-A, 5′-AGTACGCCAACAACCTGGAG-3′ , and AAGAGCTGTAAAGCCCACGA-3′; Oct-4, 5′-GGATGCTGTGAGCCAAGG-3′, and 5′-GAACAAAATGATGAGTGACAGACAG-3′ ; Nanog, 5′-CACCCACCCATGCTAGTCTT-3′ and 5′-ACCCTCAAACTCCTGGTCCT-3′ ; Ets-1, 5′-CATATCAGGTTAATGGAGCC-3′ and 5′-GTAGTCGAAGCTGTCATAGG-3′ ; GABA_A_R α1, 5′-TTTGGGAGAGCGTGTAACTGAA-3′ and 5′-ACTCCATATCGTGGTCTGAAACTG-3′; GABA_A_R β3, 5′-AATCAAAATCCCTGATCTAACCGA-3′ and 5′-AAGAGAGAAAAGGTGAATGGAAACA-3′; NPR-B, 5′-TCATGACAGCCCATGGGAAA-3′ and 5′-GGTGACAATGCAGATGTTGG-3′; β-actin, 5′-TGGTGGGCATGGGTCAGAAGGATTC-3′ and 5′-CATGGCTGGGGTGTTGAAGGTCTCA-3′.

### RNA Interference

RNA interference in murine ES cells was carried out according to the manufacturer's protocol using Lipofectamine 2000 (Invitrogen) in 6-well plates. Two pairs of siRNAs (Invitrogen) were designed for BNP (NM_008726) using the BLOCK-iT RNAi Designer software. BNP siRNA1 gave higher level of BNP knockdown and therefore used for all subsequent experiments. The appropriate siRNA negative control Duplex (Cat. No. 12935-300; Invitrogen) was selected based on the percentage G/C. The BNP siRNA and control siRNA were transfected at a final concentration of 40 µM for 24 h in triplicate for each treatment. At 48 h post-transfection, BNP knockdown was confirmed by RT-PCR and Western blotting. The sequences of BNP siRNAs were as follows:

BNP siRNA1: sense, 5′-CCCAGAGACAGCUCUUGAATT-3′; antisense, 5′- UUCAAGAGCUGUCUCUGGGTT-3′.

BNP siRNA2: sense, 5′-GGCACAAGAUAGACCGGAUTT-3′; antisense, 5′- AUCCGGUCUAUCUUGUGCCTT-3′.

### Western Blotting

Total protein extracts were prepared from ES cells, dissolved in SDS-PAGE buffer, and transferred to nitrocellulose membranes (Amersham Biosciences, Germany). Proteins were detected using antibodies against BNP (1∶2000; Chemicon), γ-H2AX (1: 1000; Upstate), phospho-Akt (Ser 473) (1: 1000, Cell Signaling), and β-actin (1∶6000, sc-47778; Santa Cruz Biotechnology). The secondary antibodies were: Histofine anti-rabbit IgG complex or Histofine anti-mouse IgG complex (1∶50; Nichirei, Japan). The blots were developed using the SuperSignal West Pico Chemiluminescent substrate (Pierce), and visualized using a LAS-3000 FujiFilm Lumino-Image Analyzer (FujiFilm, Tokyo, Japan).

### Flow Cytometry

Cells were fixed in 4% paraformaldehyde for 15 min at RT, and washed with PBS that contained 2% FBS. Cells were incubated for 3 min in 100% ethanol, and blocked in 2% BSA for 20 min at RT. For staining for Oct-4 or double-labeling for BNP and Oct-4 or NPR-A and Oct-4, the cells were incubated for 1 h on ice with the following primary antibodies: anti-BNP (1∶1000; Chemicon); anti-NPR-A (1∶200; Abcam); and anti-Oct-4 (1∶200; Santa Cruz Biotechnology). This was followed by incubation for 1 h at RT with Alexa Fluor 488-conjugated anti-rabbit antibody and/or Alexa Fluor 647-conjugated anti-mouse antibody (1∶200; Molecular Probes). Data acquisition was performed using a FACSCalibur (BD Biosciences) and the data were analyzed using the CellQuest program. At least 10,000 events were collected for each sample.

#### Cell cycle analysis

The cells were fixed overnight in 70% ethanol at 4°C. Enzymatic removal of RNA was carried out using 100 µg/ml RNase (Boehringer Mannheim GmbH, Mannheim, Germany) at RT for 20 min. The cells were then stained with 5 µg/ml propidium iodide (PI; Sigma) at 4°C for 40 min. Flow cytometric analysis was carried out on 20,000 gated events. The cell cycle phase distribution was analyzed using the MODFIT-LT Flow Cytometry Modeling Software (Verity Software House).

#### Bromodeoxyuridine (BrdU) incorporation

The level of BrdU incorporation was measured together with the DNA content. ES cells 48 h after siRNA transfection were pulsed (45 min) with BrdU (1∶100; Invitrogen). ES cells were dispersed into single cells; the cells were fixed overnight in 70% ethanol at 4°C. DNA denaturation was subsequently performed by incubation in 1 N HCl for 20 min at room temperature. The cells then washed and incubated with 0.1 M sodium tetraborate for 10 min at room temperature. The cells were incubated with Alexa Fluor 488-conjugated mouse anti-BrdU antibody (1∶100; Molecular Probes) in 2% BSA-PBS for 2 h at 4°C. The cells were then incubated with 100 µg/ml RNase (Boehringer Mannheim) for 15 min, followed by 40 min of incubation in freshly prepared PI (5 µg/ml).At least 10,000 cells events were recorded for each sample using a FACSCalibur (BD Biosciences) and analyzed using the CellQuest program. The samples were subjected to two-parameter dot plot histogram analysis (BrdU incorporation vs. DNA content).

### Alkaline Phosphatase (ALP) Activity Assay

Staining for alkaline phosphatase (ALP) was performed at room temperature using an alkaline phosphatase kit (Chemicon), according to the manufacturer's instructions. During reaction, culture dishes were protected from drying and direct light. Images were observed under an inverted microscope (Nikon, Japan).

### Cell Proliferation Assay

In order to determine the number of cells, the cells were washed with PBS and trypsinized from the culture dishes. The cell suspension was mixed with trypan blue solution, and the number of live cells was determined using a homocytometer. Cells failing to exclude the dye were considered nonviable.

### Apoptosis Assay

Annexin V/propidium iodide (PI) staining was performed using flow cyometry according to the manufacturer's guidelines (MBL, USA). Briefly, ES cells were washed in ice-cold PBS. The cells were then resuspended in 500 µl of binding buffer and incubated with 5 µl of Annexin V-EGFP and 5 µl of PI for 10 min in the dark at room temperature. Flow cytometric analysis was immediately performed using a FACSCalibur (BD Biosciences)**.**

### Intracellular cGMP Assay

Cells were lyzed with 0.1 M HCl containing 0.1% Triton X-100 at RT for 20 min. The lysates were centrifuged, and the levels of cGMP in the supernatants were measured with a cGMP enzyme immunoassay kit (Assay Designs).

### Statistical Analysis

The results are expressed as mean s.d., as indicated in the figure legends. Statistical significance was determined using the paired Student's *t*-test.

## Supporting Information

Figure S1 Exogenous BNP enhances ES cell proliferation without affecting ES cell pluripotency. [A] ES cell colonies 3 days after exposure to BNP. ES cells were supplemented with 1 µM BNP daily for 3 days in low-density cell cultures. [B] The alkaline phosphatase activities of the ES cells were measured after 4 days in the presence or absence of 1 µM BNP. Scale bars: A, 100 µm; B, 10 µm.
(0.08 MB PDF)

Figure S2 Effect of BNP signaling on phosphoinositide 3-kinase (PI3K). [A] Western blot analysis for phosphor-Akt (Ser 473) 48 h after siRNA transfection. [B] Western blot analysis for phosphor-Akt (Ser 473) after 24 h exposure to BNP (1 µM). [C] Flow cytometric analysis of phosphor-Akt (Ser 473) 48 h after siRNA transfection.
(0.16 MB PDF)
