# Supplementary figures and images for: BNP Signaling Is Crucial For Embryonic Stem Cell Proliferation
Source: PLoS One. 2009 Apr 28;4(4):e5341. doi: 10.1371/journal.pone.0005341 (PMC2670516; doi:10.1371/journal.pone.0005341)

Figure S1

**A**

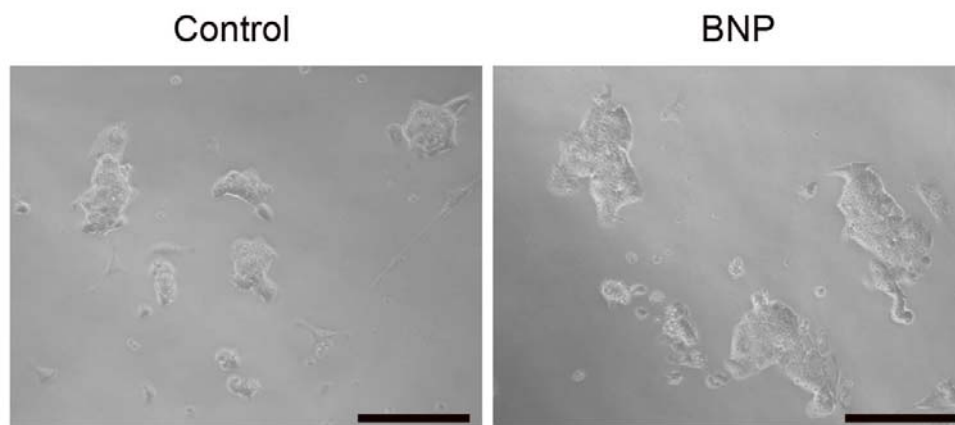

**B**

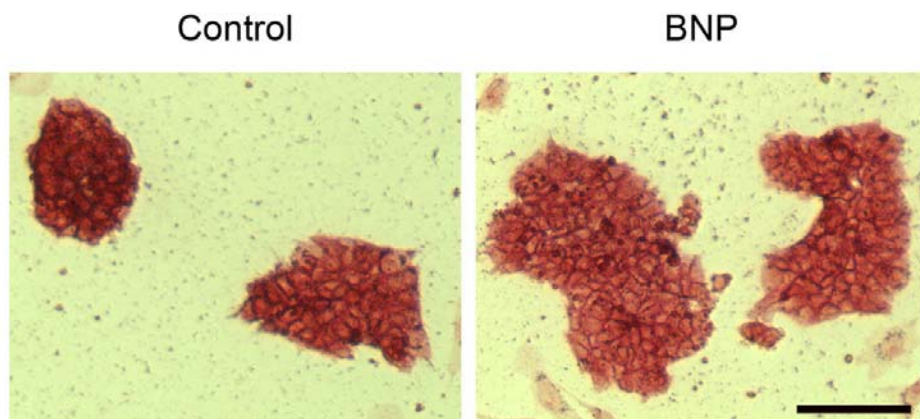

Supplement: Figure S1 — Exogenous BNP enhances ES cell proliferation without affecting ES cell pluripotency. [A] ES cell colonies 3 days after exposure to BNP. ES cells were supplemented with 1 µM BNP daily for 3 days in low-density cell cultures. [B] The alkaline phosphatase activities of the ES cells were measured after 4 days in the presence or absence of 1 µM BNP. Scale bars: A, 100 µm; B, 10 µm. (0.08 MB PDF) [file pone.0005341.s001.pdf]

Figure S2

**A**

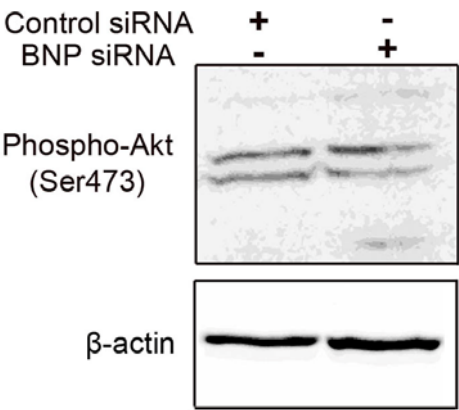

**B**

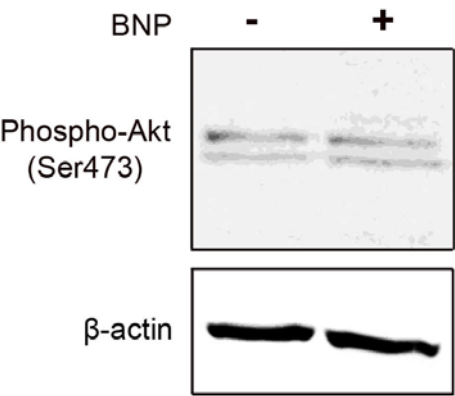

**C**

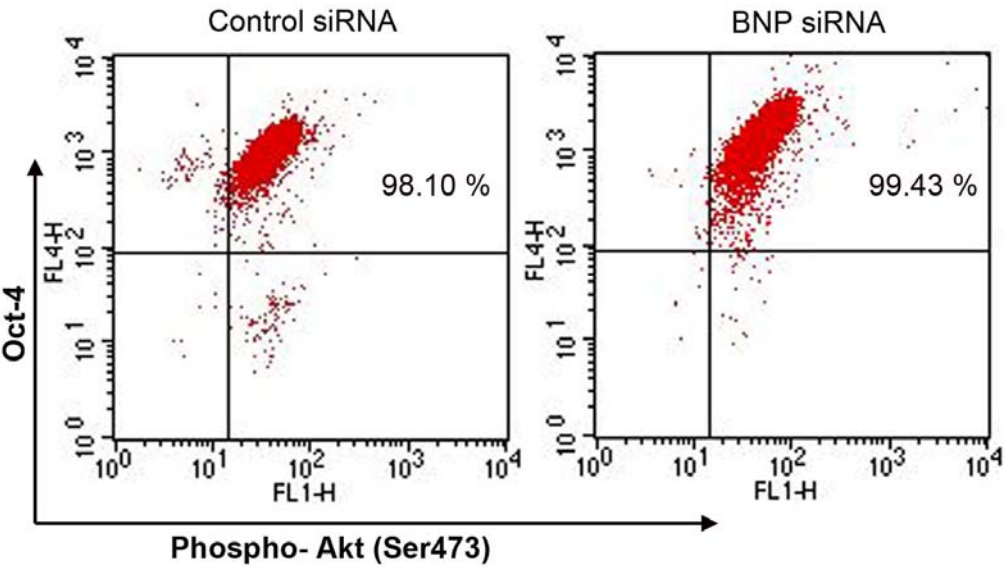

Supplement: Figure S2 — Effect of BNP signaling on phosphoinositide 3-kinase (PI3K). [A] Western blot analysis for phosphor-Akt (Ser 473) 48 h after siRNA transfection. [B] Western blot analysis for phosphor-Akt (Ser 473) after 24 h exposure to BNP (1 µM). [C] Flow cytometric analysis of phosphor-Akt (Ser 473) 48 h after siRNA transfection. (0.16 MB PDF) [file pone.0005341.s002.pdf]
